# Flexible assessment of biosecurity in small- and medium scale poultry farms in low and middle income countries

**DOI:** 10.1186/s13028-025-00796-8

**Published:** 2025-02-08

**Authors:** Søren Saxmose Nielsen, Naomi P. Kemunto, Dishon M. Muloi, Anders Miki Bojesen, Theodore Knight-Jones, Dreck Ayebare, Michel Dione, Eugine L. Ibayi, Louise Ladefoged Poulsen, Arshnee Moodley

**Affiliations:** 1 Department of Veterinary and Animal Sciences, University of Copenhagen, Grønnegårdsvej 8, 1870 Frederiksberg C, Denmark; 2 Animal and Human Health Department, International Livestock Research Institute, Nairobi, Kenya; 3 Institute of Infection, Veterinary and Ecological Sciences, University of Liverpool, Neston, UK

**Keywords:** Context-specific biosecurity, Fit-for-purpose biosecurity, LMIC, Poultry, Risk management, Risk mitigation

## Abstract

**Background:**

Biosecurity measures are essential for mitigating the risk of pathogen introduction and spread in farms. While standardised tools for monitoring biosecurity implementation exist, they are often not tailored to the specific needs of low and middle income countries (LMICs), where pathogen occurrence and farming practices can be highly variable compared to intensive high income country settings. The aim of our study was to develop a flexible risk assessment tool for evaluating biosecurity practices on small and medium-scale poultry farms in LMICs. The methodology described here allows local experts to adapt the tool to current conditions.

**Results:**

The development process began by combining two existing questionnaires. These were evaluated by nine experts with expertise in diverse farming systems in LMICs. The experts conducted a knowledge aggregation process to assign weights to the different areas and individual questions within the questionnaires. The median scores from the final expert elicitation informed the weighting of questions in the newly developed questionnaire. These weights are adaptable and can be adjusted to reflect population-specific conditions, which may vary in pathogen load and farming practices.

**Conclusions:**

We have developed a flexible biosecurity assessment tool tailored to small-and medium-scaled poultry farms in LMICs. This tool can be used as presented or adapted to local conditions through the input of local experts, allowing for effective and context-specific biosecurity monitoring.

**Supplementary Information:**

The online version contains supplementary material available at 10.1186/s13028-025-00796-8.

## Background

Biosecurity measures are essential to mitigate the risk of pathogen introduction and spread in animal production facilities. The importance of biosecurity is underscored in regulatory frameworks, such as the European Union’s Animal Health Law, where “biosecurity” is mentioned 70 times [1]. The Animal Health Law defines “*‘biosecurity’ means the sum of management and physical measures designed to reduce the risk of the introduction, development and spread of diseases to, from and within: (a) an animal population, or (b) an establishment, zone, compartment, means of transport or any other facilities, premises or location*”. Similarly, the World Organisation for Animal Health dedicated an entire chapter to biosecurity in poultry production [2], and the Food and Agricultural Organization (FAO) has developed a comprehensive biosecurity toolkit [3]. Despite these extensive resources, implementing effective biosecurity measures remains a challenge, particularly in low and middle income countries (LMICs) [4–8]. The difficulties in establishing biosecurity practices are multifaceted. On the one hand, biosecurity measures are, also by farmers, considered essential for preventing outbreaks of infectious diseases that can severely impact animal health, productivity and increased use of antimicrobials [9–11]. On the other hand, there are numerous barriers to compliance. These barriers include insufficient knowledge about the importance and implementation of biosecurity measures, high investment costs, inconvenience, and the perceived absence of immediate hazards [10–12]. Additionally, some measures may be ineffective or not evidence-based, further complicating adherence [4–6, 13].

On farm biosecurity can be conceptualised through a tiered approach: primary biosecurity focuses on reducing the risk of pathogen introduction to the farm; secondary biosecurity aims to minimise the spread of pathogens within the farm. Furthermore, increasing the resilience of farm animals through improved feeding practices or selective breeding for increased resistance to infections can complement biosecurity measures. The latter can be achieved through conventional methods targeting resistance to specific pathogens or through novel approaches like host genome editing to enhance host immunity [14–16]. Establishing appropriate measures often requires deductive research, such as risk factor studies, which systematically identify and validate factors associated with pathogen introduction and spread. However, in many cases, inductive reasoning is employed due to the lack of specific deductive information. This approach involves retaining certain biosecurity measures based on previous studies or logical assumptions, even if they have not been conclusively proven to be effective [17–19]. The challenges in designing fit for purpose risk factor studies include low prevalence of target pathogens, variable infection periods, farm heterogeneity, and residual confounding factors, all of which complicate the establishment of clear associations between risk factors and pathogen occurrence.

Formal on-farm biosecurity assessment can be conducted using a variety of tools, such as the broiler tool from Biocheck.Ugent©, which aims to describe the overall biosecurity situation in broiler flocks and is designed for assessments in commercial holdings in developed farming systems [20]. This tool has also been used in an LMIC, but for flock sizes ranging from 1300 to 900,000 [21], and not on small-and medium-scale farms. Furthermore, tools like Biocheck.Ugent© are not disease-specific and may not adequately address unique challenges posed by local settings and certain pathogens particular diseases [19, 22]. Other tools have also been used in LMICs to quantify biosecurity [23], but they lack the weighting of scores to reflect their relevance in specific contexts. Each farm, region, or country must consider the risk of introduction and the risk of spread for specific pathogens that influence both the probability of disease introduction and the consequences of an outbreak. The occurrence of pathogens—whether absent, sporadic, epidemic, or endemic—can vary significantly between farms and locations. The impact of these disease may be of concern to individual farmers, regional stakeholders or national veterinary authorities, depending on the disease category [1]. Therefore, biosecurity assessment must be tailored to local conditions to be truly effective.

The purpose of this study was to develop a flexible tool for assessing biosecurity measures in small and medium-scale poultry farms in Kenya, applicable in similar LMIC country contexts. These settings often have distinct pathogen profiles and farming practices that differ from those in high-income countries, necessitating a customised approach to biosecurity. The tool aims to provide a targeted assessment that considers the local occurrence of specific pathogens and farming conditions, allowing for a more effective and context-specific biosecurity strategy compared to LMICs of standardised tools. Such a tool is needed to allow meaningful assessment of biosecurity status and investigation of causes and consequences of variation in biosecurity in diverse settings.

## Methods

The development of the biosecurity assessment tool involved a structured four-step process that can be adapted for specific populations if necessary. The steps are: (1) selection of experts; (2) development and review of research protocol; (3) individual weighting of questions by experts; and (4) expert knowledge aggregation. Below, is a detailed description of the process used in our scenario. The target farms are those of FAO sector Classifications 2 and 3 [24] i.e. farms with roofed housing of birds, water and feed facilities, bins for dead animals and other basic infrastructure that enable to respond to the questions in the tool (see Supplementary Materials). These farms are generally below a certain size, e.g. fewer than 2000 birds per production cycle, and do not include backyard poultry. The target farms are typically located in LMICs [25].

### Step 1. Selection of experts

The selection of experts was carried out to ensure a diverse range of expertise relevant to the project. The core group of 10 experts consisted of the principal investigators, including PhD student NPK (Kenya), under the supervision of DMM (Kenya), AM (Kenya/Denmark) and SSN (Denmark). NPK, DMM and SSN are trained as veterinarians, while AM is trained in microbiology. Also, ELI (Kenya) and DA (Uganda), who are both trained as veterinarians with extensive field experience with poultry farming in LMICs, were included. The team also comprised TK-J (Ethiopia/Tanzania/Turkey/United Kingdom/Zambia), MD (Burkina Faso, Senegal and Uganda), LLP (Denmark/Vietnam) and MB (Denmark/Georgia), who are experts in poultry diseases with additional insights in the LMIC context (experience in countries mentioned).

### Step 2. Development and review of protocol

The initial protocol was drafted by SSN and circulated among the selected experts for review and feedback. This review process focused on ensuring clarity and relevance to the study objectives. After incorporating the experts’ comments, a revised version of the protocol was distributed for final approval.

### Step 3. Individual weighting of questions and provision of replacement questions

To develop the questionnaire, we combined two existing tools: a broiler farm assessment questionnaire developed at ILRI (Supplementary materials A, [26]) and the Biocheck.UGent© questionnaire (Supplementary materials B). The ILRI questionnaire served as the foundation, with the Biocheck.UGent© structure providing inspiration for additional or replacement questions. The structure from the Biocheck.UGent© questionnaire was used; this is organized into 11 areas: A. Purchase of 1-day-old chicks; B. Depopulation of broilers (slaughterhouses, traders, individuals); C. Feed and water; D. Removal of manure and carcasses; E. Visitors and farmworkers; F. Material supply; G. Infrastructure and biological vectors; H. Location of the farm; I. Disease management; J. Cleaning and disinfection; K. Materials and measures between compartments, which were originally weighted as described elsewhere [20].

Experts were asked to individually assign weights to these areas distributing 100 points across them. They then allocated points to each question within an area based on its perceived importance. For example, if 15 points were given to Area A, these points were distributed among the five sub-questions in that area. A score of 0 indicated that the question should be omitted. If a question was unclear or insufficient, experts were encouraged to suggest revisions or additional questions. The coordinator and facilitator (SSN) did not provide points.

If more than 50% of the experts voted for revising a question suggesting that there was a problem with the question formulation, it was revised accordingly by SSN, who synthesised the feedback. Questions with 50% or fewer votes for revision were retained in their original form from the Biocheck.UGent© broiler biosecurity protocol. Next, the points allocated to each question, which reflected its importance for good biosecurity, were then sub-allocated to the answer options for that question in proportion to each option’s relative effectiveness at maintaining good biosecurity. The maximum points that could be assigned to an answer option were equal to the total points allocated to the corresponding question. For instance, if an expert deemed the question "Is the farm fenced externally?" to be worth 2 points, they might have distributed those points among the answer options as follows. (i) “No” = 0 points, (ii) “Yes, partly” = 0.5 points and (3) “Yes, completely” = 2 points, with more points reflecting better biosecurity. Following the individual expert scoring, the coordinator then summarised the scores. If a consensus was reached, meaning all but two experts provide identical scores for all options within a question, further discussion of that question was deemed unnecessary.

### Step 4. Expert knowledge aggregation

An online workshop was held to discuss and resolve any questions where consensus had not been reached. All experts participated in this workshop, where the primary objective was to achieve consensus on the weighting of each question. During the workshop, experts presented the rationale behind their scores, facilitating a deeper understanding of the reasoning behind each expert’s assessment, and thus potentially aggregation around a score. If consensus could not be achieved, the median score was used. The final scores, i.e. the median of the scores from the experts following the expert knowledge aggregation, were then summarised for each biosecurity area, providing an assessment of both external and internal biosecurity separately, as well as an overall evaluation of farm biosecurity.

## Results

The final scores for the 11 biosecurity areas, determined through expert discussions, are presented in Table 1. The final questionnaire, including the median scores assigned to each question is shown in Table 2. Among all areas, “Purchase of 1-day-old chicks” (Area A), was considered the most critical, receiving 15% of the weight, while “Location of the farm” (Area H) was deemed least important, with a weight of 5%. For Area A, a score of 6.5% was assigned if day-old-chicks were self-produced. The rationale behind this score was that self-production allows for better control over the quality of the day-old-chicks, as it eliminated the uncertainties associated with transportation under unknown conditions. In contrast, practices that increase the entry of external personnel generally resulted in lower scores. For example, in Area B, the question “Who does the slaughtering”, received lower scores if external staff were involved. However, the presence of procedures to mitigate risks associated with external personnel led to higher scores, highlighting the importance of risk management strategies. In some cases, such as the question “Which disinfectant do you use?” the response options were tailored to availability of brands familiar to farmers. The weights assigned to these options were based on the contents and perceived effectiveness of these products. This approach ensures that the questionnaire remains relevant to the local context, while maintaining a focus on effective biosecurity practices.

**Table 1:** Area-wise weighting of biosecurity

Area	%
A. Purchase of 1-day-old chicks	15
B. Depopulation of broilers (slaughterhouses, traders, individuals)	7
C. Feed and water	8
D. Removal of manure and carcasses	7
E. Visitors and farmworkers	12
F. Material supply	7
G. Infrastructure and biological vectors	10
H. Location of the farm	5
I. Disease management	10
J. Cleaning and disinfection	13
K. Materials and measures between compartments	6
Sum	100

Weighting of biosecurity scores by area based on scores from nine experts with expertise in broiler farm biosecurity in low and middle income countries

**Table 2:** Questionnaire and weights

Area question	Levels	Weight	Score
**A. Purchase of 1-day-old chicks**	Levels	**15**	
How did you acquire these birds?		6.5	
	Purchased		0
	Self-produced		6.5
Are your 1-day-old chicks (during the last 2 years) always bought from the same original source?		2	
	Always the same supplier		2
	Sometimes a different supplier		0
How often a year are 1-day-old chicks delivered to your farm?		2	
	Less than 3 times a year		2
	Between 3 and 6 times a year		1
	More than 6 times a year		0
Are specific quality demands made for the supplier of day-old-chicks?		4.5	
	Yes, multiple		4.5
	Yes, some		2
	No, they are purchased with no specific demands		0
Which specific quality demands are made for the supplier		0	*
	Specific pathogen free		
	Vaccinated		
	Mortality below specific threshold		
	Temperature requirements during transport		
	Requirements for maximum transport duration		
**B. Depopulation of broilers (slaughterhouses, traders, individuals)**		**7**	
Are broilers slaughtered on farm or transported to other sites?		1	
	Slaughtered on farm		1
	Transported to another site		0
Who does the slaughtering		2	
	Farmer alone or with own staff		2
	Farmer with staff from outside		0
	Done elsewhere		1
Does all-in-all-out production occur on farm-level		2	
	Yes		2
	No		0
Does all-in-all-out production occur on coup-level?		2	
	Yes		2
	No		0
**C. Feed and water**		**8**	
Is there an automated water delivery system?		0.5	
	Yes		0.5
	No		0
What is the source of drinking water for chickens?		2	
	Dam		0.3
	River		0.3
	Tap water		1.5
	Borehole		1
	Rain water		1
	Water vendor		1
	Municipal		1.5
Is the water used for the chickens treated for the birds?		1	
	Yes		1
	No		0
What do you use in the treatment?		0.9	
	Alum		0.5
	Flocculant		0.5
	Chlorine		0.9
Where do you store the feed?		1	
	I use all		0.6
	In separate room		0.8
	In same room		0.3
	Outside		0.1
	Other		0.1
How do you store the feed?		1.1	
	On floor		0
	Elevated		1.1
Are the feed/concentrate storage rooms (areas) completely sealed against water, birds and vermin?		1.5	
	Yes		1.5
	No		0
**D. Removal of manure and carcasses**		**7**	
Is there an observed separate area (outside chicken house) to store carcasses (dead birds)		1	
	Yes		1
	No		0
Is the carcass storage area protected from vermin, cats and/or dogs?		1.7	
	No		0
	Yes, partly		0.4
	Yes, completely		1.7
Do you take any measures to keep the carcass storage area clean?		1.1	
	None		0
	Sweep		0.5
	Clean with water		0.4
	Disinfect		0.9
How often do you take these measures?		0.9	
	Never		0
	After use		0.9
Is the carcass storage space/area cleaned and disinfected after each use?		1.3	
	Never		0
	Sometimes		0.4
	Always		1
Is the manure removed and disposed of appropriately through the dirty road?		1	
	Yes		1
	No		0
**E. Visitors and farmworkers**		**12**	
Is there a functional disinfection foot bath at the entrance of the farm?		1.2	
	Yes		1.2
	No		0
Which disinfectant do you use?		0.5	
	Norocleanse		0.5
	Biosafe		0.5
	Jik		0.3
How often do you change the fluid?		1	
	> Weekly		0
	Weekly		0.5
	Daily		0.9
Do you take any measures to ensure workers who enter the poultry house do not transfer any disease to the poultry?		2	
	None		0
	Wash hands		0.5
	Wear PPE		1
	Disinfect hands		1
Is the farmer able to show you a handwashing facility		0.4	
	Yes		0.4
	No		0
Are you able to observe the disinfectant?		0.3	
	Yes		0.3
	No		0
Are employees required to wear specific clothing before they are allowed to enter the poultry houses?		0.8	
	Yes		0.8
	No		0
Do farm employees have to wash and disinfect their hands before they are allowed to enter the poultry houses?		0.5	
	Yes		0.5
	No		0
Are there any employees who also keep poultry or any other type of bird at their own home?		0.8	
	Yes		0
	No		0.8
Are there any employees who also work on other poultry farms?		0.8	
	Yes		0
	No		0.8
Do non-farm personnel (such as traders or veterinarians) sometimes enter the poultry houses?		0.7	
	Yes		0
	No		0.7
Do you have specific procedures in place in case a visitor must enter the poultry houses?		0.8	
	Cannot enter		0.8
	Yes		0.5
	No		0
What are the specific procedures in place in case a visitor must enter the poultry houses?		2.2	
	None		0
	Wash		0.5
	Disinfect footwear		0.5
	Farm specific shoes		0.8
	Footbath		0.5
	Not allowed to enter		2.2
	Notify farm manager		0.4
	Wear PPE		0.6
	Other		0
**F. Material supply**		**7**	
Is there any material being shared with other farms that enters the poultry houses and/or has contact with your poultry?		3.5	
	Yes		3.5
	No		0
Are specific measures taken for the introduction of material (e.g. UV-disinfection unit, alcohol disinfection)? (		3.5	
	Yes		3.5
	No		0
**G. Infrastructure and biological vectors**		**10**	
How are the chickens housed?		1.7	
	Outdoor		0
	Outdoor access		0
	Indoor		1.7
Is the farm fenced externally?		1.6	
	No		0
	Yes, partly		0.5
	Yes, completely		1.6
Can wild birds enter the poultry house?		1.6	
	Yes		0
	No		1.6
Have you ever seen wild birds in the poultry house?		0.8	
	Yes		0
	No		0.8
Are vermin (rats, mice etc.) considered a problem on the farm?		1.3	
	Never		1.3
	Often		0
	Sometimes		0.5
Are there pets (dogs and cats) on the farm?		0.6	
	None		0.6
	Cats		0
	Dogs		0
	Other		0
Can the pets access the poultry house?		1.3	
	Yes		0
	No		1.3
	na		1.3
Are “backyard” chickens also kept on the farm premises?		1.1	
	Yes		0
	No		1.1
**H. Location of the farm**		**5**	
What is the approximate distance to the nearest neighbouring poultry farm?		2	
	< 500 m		0
	500–1000 m		1
	> 1000 m		2
Is manure from other poultry farms spread on the neighbouring farmlands (within a 500 m (0.3 miles) radius)?		2	
	Often		0
	Sometimes		1
	Never		2
Is neighbouring farms with animals fenced effectively off (to avoid animal traffic)		1	
	No neighbouring farm		1
	Yes		1
	No		0
**I. Disease management**		**10**	
On average, how many birds can be housed in each room/coop?		1	
	> 2000		0.2
	500–2000		0.4
	200–499		0.5
	100–199		0.7
	50–99		0.8
	< 50		1
		0.3	
	Never		0
	Sometimes		0.1
	Always		0.3
Is there a separate area for diseased birds?		1.5	
	Yes		1.5
	No		0
Do you take any measures to keep area for diseased birds clean?		0.9	
	None		0
	Sweep		0.3
	Clean with H20		0.3
	Disinfect		0.3
	Other		0
How often do you normally take measures to keep area for diseased birds clean?		0.7	
	After use		0.7
	Monthly		0.1
Did you know if your birds had been vaccinated when your received them?		1.5	
	Yes		1.5
	No		0
Which vaccines had they been given?		1.3	
	Not vaccinated		0
	IB		1.2
	IBD		0.8
	Newcastle		1
	Mareks		0.2
	Vaccinated but not known with which vaccines		0.7
Have you vaccinated since then?		0.9	
	Yes		0.9
	No		0
Which vaccines did you administer?		0.9	
	Not vaccinated		0
	IB		0.7
	IBD		0.7
	Newcastle		0.7
	Mareks		0.2
Who administered the vaccines?		0.2	
	Farm owner		0.2
	Farm manager		0.2
	Other staff		0.2
Why did you give the vaccines?		0.8	
	General disease prevention		0.8
	Previous outbreak		0.3
	Advised to do so		0.2
**J. Cleaning and disinfection**		**13**	
Are vehicle disinfection baths(channels) available at the entrance of the farm?		0.5	
	Yes		0.5
	No		0
Is there a functional foot bath at the entrance of each chicken coop/house?		1.4	
	Yes		1.4
	No		0
How often do you change this disinfectant?		1	
	Never		0
	Monthly		0.2
	Weekly		1
Do the workers disinfect before they enter the poultry house?		1.5	
	Yes		1.5
	No		0
What do they use when disinfecting?		0.3	
	Bassin		0.1
	Hand-held sprayer		0.3
Is there a protocol for the cleaning and disinfection of drinkers after each production cycle?		0.9	
	Yes		0.9
	No		0
Is there a protocol for the cleaning and disinfection of feeders after each production cycle?		0.9	
	Yes		0.9
	No		0
How long (in days) is the resting period between production cycles?		2	
	0–7 days		0
	8–14 days		0.2
	15–21 days		0.2
	> 22 days		2
Do you take any measures to keep the feed storage area clean?		1	
	No feed storage		0.4
	None		0
	Sweep		0.3
	Clean with H20		0.5
	Disinfect		0.7
How often do you normally do this?		0.5	
	Never		0
	Quarterly		0
	Monthly		0.1
	weekly		0.5
Is the farm divided into clean and dirty area?		1	
	Yes		1
	No		0
How do you clean your poultry house after a flock is sold		2	
	Remove waste		0.6
	Disinfect		0.8
	Clean feeders		0.2
	Disinfect feeders		0.4
	Other		0.3
**K. Materials and measures between compartments**		**6**	
How many separate houses are total?		2	
	1		2
	2–5		0.8
	> 5		0.1
How many separate rooms/coops are there in total (in all structures)?		2	
	1		2
	2–5		0.8
	> 5		0.1
Has clearly recognisable, separate material been foreseen for each poultry house?		2	
	Yes		2
	No		0

Final questionnaire and weighting scores from nine experts with expertise in broiler farm biosecurity in low and middle income countries

Text in bold font indicates “Areas” and the weighting of the area-levels. Text in normal font are question-specific and the weight of the questions within the area

^*^ No weight for this question, but in principle linked to the previous question, which the present question can be used to inform

## Discussion

There is growing evidence that raising awareness and improving biosecurity measures can encourage the prudent use of antibiotics, and that biosecurity practices, coupled with vaccination could partially replace antibiotic use as productivity-enhancing and disease management tools in broiler farms in Senegal [27]. In this study, we developed a questionnaire tailored for assessing biosecurity measures in broiler farms in LMICs. The weighting of biosecurity measures for the different questionnaire areas and questions have been developed by a panel of ten experts based on their understanding of small and medium-scale poultry farms in LMICs. This is an improvement over previous tools used in LMICs [21, 23, 26], which have quantified biosecurity but lacked either weighted scoring or transparency in the weighting process. However, these scores should not be considered applicable to all other settings or poultry production systems e.g. layers, and we demonstrate how the questionnaire can be recalibrated for different settings. This will enable veterinary authorities or other stakeholders to adapt the assessment tool for local interpretation and to inform the development of interventions that are tailored to specific settings, including local populations and pathogens. It is important to note that this process can be challenging, as it requires those developing the questionnaire to include a discussion on the target pathogens.

Our approach demonstrates the process for developing such a tool, but it is crucial to recognise that the scores are not universally applicable. Local assessments should be conducted to ensure that the tool is adapted to the specific conditions, including the local population and prevalent pathogens. For example, this could involve evaluating the association between biosecurity measures and the occurrence of specific pathogens, such as *Campylobacter* spp. [22]. Moreover, understanding the combination of purpose, intensity and production level of broiler production provides a specific context for the use of the biosecurity tool. For example, small-scale farmers might be more willing to accept risks associated with visitors due to the lower stakes compared to large industrial farms. Additionally, challenges in quality control, such as transport conditions or the age of day-old chicks, may limit the precision and utility of certain questions if farmers lack control over these variables. Despite these limitations, improving biosecurity through better chick quality assessment can have a significant impact on immediate and long-term flock health, as poor quality can lead to the carriage and eventual transmission of infectious agents. Developing more specific parameters to effectively monitor the quality of day-old chicks is therefore essential. Moreover, knowledge of pathogen circulation in the area can lead to more targeted questions on cleaning, disinfection, vaccination strategies, and risks associated with wildlife exposure. Such information would be invaluable for experts when scoring, ensuring a more informed and unified approach.

The weighting of different areas can be discussed. For instance, we assigned an overall weight of 7% to depopulation (Section B), compared to 11% assigned in an industrialised context [20]. These and other weighting differences are important to note, as they may reflect relative importance based on specific contexts rather than absolute importance. In general, all tools discourage the entrance of visitors, such as external staff [20–23, 28]. However, in small and medium-scale farms in LMICs, external staff may not be necessary for depopulation, as farmers often handle the removal of birds themselves. Such differences in practices can influence the relative weights assigned to the various areas of biosecurity.

Whilst most experts in our study had a Kenya-centric perspective, we included individuals with broader expertise across Eastern Africa and other countries, such as Burkina Faso, Senegal, Georgia, and Vietnam. This approach provided a nuanced perspective and allowed us to incorporate a wide range of expertise; however, due to the diversity of the expert panel, we were unable to fully tailor the tool to a specific local population. In many cases, it may be challenging to find experts with deep local knowledge, which can introduce biases in the weighting process. During the planning phase, it is essential to weight the pros and cons of expert selection carefully. Producers’ attitudes toward biosecurity vary significantly [29], and their motivation is important in the risk assessment process. Engaging in active discussion with producers can not only serve both an educational purpose but also foster greater acceptance of the necessary changes to daily routines for improved biosecurity [30].Engaging producers has been shown to the pivotal in e.g. reducing antimicrobial use through improved biosecurity [9, 12]. Therefore, involving producers in the development of the tool could have been valuable, particularly given the time required to administer the survey i.e. we used 30–45 min per farm in a study of 19 farms. While this may seem lengthy, the process could also serve as a training opportunity to raise awareness of specific biosecurity aspects and engage farmers in the risk assessment process [28, 31]. However, the time commitment of 30–45 min can be a challenge to maintaining farmer motivation, especially if they do not perceive clear benefits. This should be carefully considered when using the tool. Despite its length, our tool includes only 72 questions, fewer than the 170 questions found in another recently developed tool [22].

We provide a flexible tool that can be adapted by local risk assessors to fit specific conditions. The development process is transparent, iterative and clearly outlined, and the weighting can be adjusted for any population, including at the farm-level. While standardised tools like Biocheck.Ugent© offer the advantage of comparability across populations, facilitating research into risk factors of pathogen introduction [5], they may not always be serve the best interests of individual farmers or local communities. Our tool can be customised to address the unique assessment requirements for local conditions and challenges faced by farmers in LMICs, making it a valuable resource for relevant biosecurity assessment and improvement. The tool can be used in various ways, such as training farmers to identify weaknesses in biosecurity measures on their farms, conducting research (e.g. comparing biosecurity measures between farms with high and low mortality or antimicrobial use), and monitoring biosecurity by competent veterinary authorities. For the latter, introducing categories of “acceptable” biosecurity as done by others [22], could be helpful. However, we have deliberately refrained from doing so, as defining such thresholds is a decision for risk managers. The selection of cut-offs should align with specific objectives such as improving food safety or increasing farming profitability.

## Conclusions

In conclusion, we developed a tool that can be used to assess the biosecurity on small- and medium-sized poultry farms in LMICs. The tool is flexible and can be modified to other specific conditions with input from local poultry experts. Such a tool is needed to identify gaps in biosecurity, and as a quantitative metric to allow further investigation of the causes, correlates and consequences of variable standards of biosecurity with view to informing strategies and policy. Such steps are needed to improve poultry productivity and welfare, and to reduce antimicrobial usage.

## Supplementary Information

Supplementary Material 1.

Supplementary Material 2.

## Data Availability

Not applicable.

## Abbreviations

FAO: Food and Agriculture Organization of the United Nations
LMIC: Low and middle income countries

## Declarations

### Prior publication

Data have not been published previously.
