# Supplementary material for: Flexible assessment of biosecurity in small- and medium scale poultry farms in low and middle income countries
Source: Acta Vet Scand. 2025 Feb 8;67:9. doi: 10.1186/s13028-025-00796-8 (PMC11806742; doi:10.1186/s13028-025-00796-8)
Supplement: Supplementary file 1 — Supplementary Material 1. [file 13028_2025_796_MOESM1_ESM.docx]

**Additional file A. Questions in the developed ILRA questionnaire.**

| **Biocheck** | **Variable** | **Question** | **Dependencies** |
| --- | --- | --- | --- |
| **C. Feed and water** | | | |
|  | s13q80_is_auto_drinkers | Is there an automated water delivery system? |  |
|  | s13q81_water_source | What is the source of drinking water for chickens? |  |
|  | s13q82_is_treated_h2o | Is the water used for the chickens treated for the birds? |  |
|  | s13q83_h2o_treatment | What do you use in the treatment? | q82 |
|  | s31q287_feed_store | Where do you store the feed? |  |
|  | s31q287_feed_store_other | Where do you store the feed other? | q287 |
|  | s31q288_store_floor | How do you store the feed? |  |
|  | s31q289_is_store_sealed | Are the feed/concentrate storage rooms (areas) completely sealed against water, birds and vermin? |  |
| **D. Removal of manure and carcasses** | | | |
|  | s19q123_is_sep_area4carcass | Is there a separate area (outside chicken house) to store carcasses (dead birds) |  |
|  | s19q124_is_see_carcass_area | Ask to see the carcass storage area. Are you able to see it? | q123 |
|  | s19q125_is_carcass_area_protected | Is the carcass storage area protected from vermin, cats and/or dogs? | q123 |
|  | s19q126_carcass_clean_measures | Do you take any measures to keep the carcass storage area clean? | q123 |
|  | s19q127_freq_carcass_clean_measures | How often do you take these measures? | q123 |
|  | s19q128_freq_carcass_disinfected | Is the carcass storage space/area cleaned and disinfected after each use? | q123 |
| **E. Visitors and farmworkers** | | | |
|  | s14q85_is_foot_bath | Is there a disinfection foot bath at the entrance of the farm? |  |
|  | s14q86_is_used_footbath | Is the footbath currently in use/functional? | q85 |
|  | s14q87_footbath_disinfectant | Which disinfectant do you use? | q85 |
|  | s14q88_footbath_disinfectant_change | How often do you change the fluid? | q85 |
|  | s17q106_measures_against_xtransfer | Do you take any measures to ensure workers who enter the poultry house do not transfer any disease to the poultry? |  |
|  | s17q107_is_show_handwash | Is the farmer able to show you a handwashing facility |  |
|  | s17q108_is_show_disinfectant | Are you able to observe the disinfectant? |  |
|  | s17q109_is_wear_ppe | Are employees required to wear specific clothing before they are allowed to enter the poultry houses? |  |
|  | s17q110_is_workers_wash_hands_b4_enter | Do farm employees have to wash and disinfect their hands before they are allowed to enter the poultry houses? |  |
|  | s17q111_is_workers_keep_poultry | Are there any employees who also keep poultry or any other type of bird at their own home? |  |
|  | s17q112_is_workers_work_elsewhere | Are there any employees who also work on other poultry farms? |  |
|  | s17q113_is_entry_by_others | Do non-farm personnel (such as traders or veterinarians) sometimes enter the poultry houses? |  |
|  | s17q114_is_visitor_entry_protocol | Do you have specific procedures in place in case a visitor must enter the poultry houses? |  |
|  | s17q115_entry_protocol | What are the specific procedures in place in case a visitor must enter the poultry houses? |  |
|  | s17q115_entry_protocol_other | What are the specific procedures in place in case a visitor must enter the poultry houses other? | q115 |
| **G. Infrastructure and biological vectors** | | | |
|  | s13q76_housing_type | How are the chickens housed? |  |
|  | s14q84_is_fenced | Is the farm fenced externally? |  |
|  | s18q117_is_wild_birds_entry | Can wild birds enter the poultry house? |  |
|  | s18q118_is_seen_wild_birds | Have you ever seen wild birds in the poultry house? | q117 |
|  | s18q119_freq_vermin_problem | Are vermin (rats, mice etc) considered a problem on the farm? |  |
|  | s18q120_is_pets | Are there pets (dogs and cats) on the farm? |  |
|  | s18q121_is_pets_access_coop | Can the pets access the poultry house? | q120 |
|  | s18q122_is_backyard_chicken | Are “backyard” chickens also kept on the farm premises? |  |
| **H. Location of the farm** | | | |
|  | s18q116_distance2near_farm | What is the approximate distance to the nearest neighbouring poultry farm? |  |
| **I. Disease management** | | | |
|  | s13q79_avg_no_coop | On average, how many birds can be housed in each room/coop? |  |
|  | s20q129_is_sep_area4diseased | Is there a separate area for diseased birds? |  |
|  | s20q130_is_see_diseased_area | Are you able to see the area for diseased birds? | q129 |
|  | s20q131_diseased_area_clean_measures | Do you take any measures to keep area for diseased birds clean? | q129 |
|  | s20q132_freq_disease_clean_measure | How often do you normally take measures to keep area for diseased birds clean? | q129 |
| **J. Cleaning and disinfection** | | | |
|  | s14q89_is_veh_bath | Are vehicle disinfection baths(channels) available at the entrance of the farm? |  |
|  | s15q93_is_coop_footbath | Is there a foot bath at the entrance of each chicken coop/house? |  |
|  | s15q94_is_used_coopbath | Is this footbath in use/functional? | q93 |
|  | s15q95_is_same_disinfectant | Do you use the same disinfectant as is the entrance footbath? | q93 |
|  | s15q96_coop_disinfectant | Which one do you use | q93 |
|  | s15q97_freq_coop_disinfection | How often do you change this disinfectant? | q93 |
|  | s15q97a_is_disinfect | Do the workers disinfect before they enter the poultry house? | q93 |
|  | s15q97b_disinfectant | What do they use when disinfecting? | q93 |
|  | s16q100_is_drinkers_clean_protocol | Is there a protocol for the cleaning and disinfection of drinkers after each production cycle? |  |
|  | s16q101_is_feeders_clean_protocol | Is there a protocol for the cleaning and disinfection of feeders after each production cycle? |  |
|  | s16q102_check_disinfection_outcome | Do you check whether your disinfection process actually works (the efficiency) after each production cycle? |  |
|  | s16q103_resting_period | How long (in days) is the resting period between production cycles? |  |
|  | s16q104_clean_feed_store | Do you take any measures to keep the feed storage area clean? |  |
|  | s16q105_freq_clean_feedstore | How often do you normally do this? | q104 |
|  | s16q98_is_farm_divided | Is the farm divided into clean and dirty area? |  |
|  | s16q99_clean_coop | How do you clean your poultry house after a flock is sold |  |
|  | s16q99_clean_coop_other | How do you clean your poultry house after a flock is sold other | q99 |
| **K. Materials and measures between compartments** | | | |
|  | s13q77_no_house_structure | How many chicken housing structures are present on this farm? |  |
|  | s13q78_no_coops | How many separate rooms / coops are there in total (in all structures)? |  |
