# Supplementary material for: Flexible assessment of biosecurity in small- and medium scale poultry farms in low and middle income countries
Source: Acta Vet Scand. 2025 Feb 8;67:9. doi: 10.1186/s13028-025-00796-8 (PMC11806742; doi:10.1186/s13028-025-00796-8)
Supplement: Supplementary file 2 — Supplementary Material 2. [file 13028_2025_796_MOESM2_ESM.docx]

**Additional file B. Questions in Biocheck.Ugent© Broilers**

| **Area** | **Question** | **Options** |
| --- | --- | --- |
| **A** | **Purchase of one-day-old chicks** |  |
|  | 1. Are your one-day-old chicks (during the last 2 years) always bought from the same original source? | Always the same supplier  Sometimes a different supplier |
|  | 2. Are the bought one-day-old chicks first delivered at your farm, i.e. before other farms are supplied by the same transport vehicle? | Always  Sometimes  Never |
|  | 3. Are the transport vehicles (including the transport crates and containers) cleaned and disinfected before the one-day-old chicks are loaded? | Yes  No |
|  | 4. How often a year are one-day-old chicks delivered to your farm? | Less than 3 times a year  Between 3 and 6 times a year  More than 6 times a year |
| **B** | **Depopulation of broilers (slaughterhouses, traders, individuals)** |  |
|  | 5. Is the transport vehicle (including the transport crates and containers) for poultry empty on arrival at the farm? | Always  Sometimes  Never |
|  | 6. Is the transport vehicle (including the transport crates and containers) for poultry always cleaned and disinfected on arrival at the farm? | Yes  No |
|  | 7. Do the driver and the catching team receive and wear farm-specific or disposable clothes/footwear during the loading of poultry? | Always  Sometimes  Never |
|  | 8. Are individuals and traders allowed to enter the poultry houses where direct contact with the poultry is possible? | Always  Sometimes  Never |
|  | 9. In how many steps does the depopulation of a poultry house take place? | In one step  In two steps  In more than two steps |
|  | 10. How often a year are broilers moved from the farm? | Less than 6 times a year  Between 6 and 12 times a year  More than 12 times a year |
| **C** | **Feed and water** |  |
|  | 11. Is the farm site divided into a clean and dirty area?  The clean road/area is the area of the production site with restricted access, i.e. this is the area where only animals from the farm,  persons after they have applied the hygienic measures in the hygiene lock, and farm-specific materials and vehicles are allowed. The  dirty area comprises all other parts of the farm where visitors, external vehicles, … have access to. The dirty area also includes the  carcass storage facility. | Yes  No  I don’t know |
|  | 12. Is there a clear separation between the clean and the dirty area of the farm premises? | Yes  No |
|  | 13. Can the feeding company fill up the silos/deliver feed without entering the clean area? | Yes  Only some of them  None |
|  | 14. Does the feed supplier have access to the houses where direct contact with the poultry is possible? | Always  Sometimes  Never |
|  | 15. Are the feed silos or the feed storage rooms (storage of complete feed or concentrate) completely sealed against water, birds and vermin? | Yes  No |
|  | 16. How often a year does the feeding company fill up the silos or deliver feed? | Less than 20 times a year  Between 20 and 35 times a year  More than 35 times a year |
|  | 17. How often are bacteriological analyses of the drinking water performed? | At least once a year  Every two years  Less frequent than every two years  Never |
|  | 18. Where are the water samples for the bacteriological analyses taken? | At the source  At the last drinker  At both locations, i.e. at the source and the last drinker |
| **D** | **Removal of manure and carcasses** |  |
|  | 19. Is the manure removed and disposed of appropriately through the dirty road?  The clean road/area is the area of the production site with restricted access, i.e. this is the area where only animals from the farm, persons after they have applied the hygienic measures in the hygiene lock, and farm-specific materials and vehicles are allowed. The dirty area comprises all other parts of the farm where visitors, external vehicles, … have access to. The dirty area also includes the carcass storage facility. | Yes  No |
|  | 20. Is there a separate carcass storage? | Yes  No |
|  | 21. Can the carcasses be collected by the rendering company without entering the farm premises (e.g. from the public road)? | Yes  No |
|  | 22. Is the carcass storage space protected from vermin, cats and/or dogs? | Yes, it's completely protected  It's only partially protected  No |
|  | 23. Is this carcass storage space cleaned and disinfected after each use? | Always  Sometimes  Never |
|  | 24. Is the carcass storage cooled? | Yes  No |
|  | 25. Are carcasses manipulated with gloves, or are hands cleaned and disinfected after manipulation of carcasses? | Always  Sometimes  Never |
| **E** | **Visitors and farmworkers** |  |
|  | 26. Are visitors obliged to notify you of their presence before entering the poultry houses (e.g. visitor's register)? | Yes  No |
|  | 27. Do all farmworkers (including the farm owner) abide by the access rules? | Always  Sometimes  Never |
|  | 28. Is a poultry-free period (longer than 12 hours) expected of all visitors before they are allowed to enter the poultry houses? | Yes  No |
|  | 29. Do visitors and farmworkers have to wear farm-specific clothing before they are allowed to enter the poultry houses? | Yes  No |
|  | 30. Do visitors and farmworkers have to wear farm-specific shoes/overshoes before they are allowed to enter the poultry houses? | Yes  No |
|  | 31. Do visitors and farmworkers have to wash and disinfect their hands before they are allowed to enter the poultry houses? | Yes  No |
|  | 32. How many times per year is access to the poultry houses granted to visitors? | Access is never granted  Access is granted, but less than 12 times a year  Access is granted more than 12 times a year |
|  | 33. Are there any farmworkers who also keep poultry or any other type of bird at home? | Yes  No |
|  | 34. Are there any farmworkers who also work on other poultry farms? | Yes  No |
| **F** | **Material supply** |  |
|  | 35. Is there any material being shared with other farms that enters the poultry houses and/or has contact with your poultry? | Yes  No |
|  | 36. Are specific measures taken for the introduction of material (e.g. UV-disinfection unit, alcohol disinfection)? | Yes  No |
| **G** | **Infrastructure and biological vectors** |  |
|  | 37. Does the poultry have access to the outside, i.e. the open air? | Yes  No |
|  | 38. Is manure being stored on the farm? | Yes  No |
|  | 39. Can wild birds enter the poultry houses? | Yes  No |
|  | 40. Are bird- and vermin-proof grids placed on the air inlets? | Yes  No |
|  | 41. Is the farm fenced? | Yes, it's completely fenced  It's only partially fenced  No |
|  | 42. Is the outside of the farm (around the walls) paved and clean (e.g. removal of weeds, waste, …)? | Yes, it's completely paved and clean  It's only partially paved and clean  No |
|  | 43. Are vermin (i.e. rats, mice, etc.) considered to be a problem on the farm? | Often  Sometimes  Never |
|  | 44. Is a rodent control programme present on the farm (other than cats)? | Yes  No |
|  | 45. Do pets have access to the poultry houses (including the hygiene lock)? | Yes  No |
|  | 46. Is “backyard”-poultry or any other type of bird being kept on the farm premises? | Yes  No |
|  | 47. Are any other farm animals being kept on the same farm site? | Yes  No |
| **H** | **Location** |  |
|  | 48. Is there stagnant or running water within a 1-kilometre radius (0.6 miles) of the farm? | Yes  No |
|  | 49. At what distance (straight-line) is the nearest neighbouring poultry farm located? | Less than 500 metres (Less than 0.3 miles)  Between 500 metres and 1 kilometre (between 0.3 and 0.6 miles)  More than 1 kilometre (more than 0.6 miles) |
|  | 50. Is manure from other poultry farms spread on the neighbouring farmlands (within a 500-metre (0.3 miles) radius)? | Often  Sometimes  Never |
|  | 51. Does animal transport frequently occur (i.e. minimum once a day) via the public road (road less than 100 metres (328 feet) from your farm) where your farm is located at (e.g. due to the location of a slaughterhouse in the neighbourhood…)? | Yes  No |
| **I** | **Disease management** |  |
|  | 52. Is there a protocol for vaccinations? If so, do you always abide by it? | Yes  No |
|  | 53. Is there a regular (i.e. at least once a year) evaluation made of the disease status of the farm (e.g. serology, trends in slaughterhouse findings, etc)? | Yes  No |
|  | 54. How often are the dead birds removed from the poultry house? | Daily  Every two days  Less frequent than once every two days |
|  | 55. What is the stocking density (according to final weight) of the poultry house? | ≤ 33 kg/m²  34 kg/m²  35 kg/m²  36 kg/m²  37 kg/m²  38 kg/m²  39 kg/m²  40 kg/m²  41 kg/m²  42 kg/m²  > 42 kg/m² |
|  | 56. Are there different age categories of poultry present on your farm? | Yes  No |
| **J** | **Cleaning and disinfection** |  |
|  | 57. Are vehicle disinfection baths or channels available at the entrance of the farm? | Yes  No |
|  | 58. Are the vehicle disinfection baths/channels always used? | Yes  No |
|  | 59. Are the poultry houses cleaned after each production cycle? | Yes  No |
|  | 60. Are the poultry houses disinfected after each production cycle? | Yes  No |
|  | 61. Is the efficacy of cleaning and disinfection checked after each production cycle (e.g. hygienogram or swabs)? | Always  Sometimes  Never |
|  | 62. Is the loading and unloading area cleaned and disinfected after each production cycle? | Yes  No |
|  | 63. How long (in days) does the sanitary break after each production cycle last? | Less than 3 days  Between 3 and 8 days  More than 8 days |
|  | 64. Is there a farm hygiene lock available? | Yes  No |
|  | 65. Is there a strict separation between the clean and the dirty area of the farm hygiene lock? | Yes  No |
|  | 66. Is there a changing room with farm-specific clothes and shoes in the farm hygiene lock? | Yes  No |
|  | 67. Is there a house hygiene lock present at every poultry house? | Yes  No |
|  | 68. Is there a strict separation between the clean and the dirty area of the house hygiene lock? | Yes  No |
|  | 69. Is there a disinfection bath/boot washer present in the house hygiene lock? | Yes  No |
|  | 70. Is it possible to wash and disinfect your hands in the house hygiene lock? | Yes  No |
|  | 71. Is there a disinfection bath/boot washer at the entrance of the farm? | Yes  No |
|  | 72. Is the fluid of the disinfection baths immediately changed when visually contaminated? | Yes  No |
|  | 73. Is the drinking water system properly cleaned and disinfected both on the in- and outside after each production cycle? | Always  Sometimes  Never |
|  | 74. Are the feeding systems properly cleaned and disinfected both on the in- and outside after each production cycle? | Always  Sometimes  Never |
|  | 75. Are the feed silos cleaned and disinfected on the inside? | Yes, after every one or two production cycle(s)  Sometimes  Never |
| **K** | **Materials and measures between compartments** |  |
|  | 76. Is there a protocol for the cleaning and disinfection of material after each production cycle and is this protocol always abided by? | Yes  No |
|  | 77. Are there multiple poultry houses present on the farm? | Yes  No |
|  | 78. Has clearly recognisable, separate material been foreseen for each poultry house? | Yes  No |
|  | 79. Are poultry house-specific clothes and boots available? | Yes  No |
